# Low-dissipation optimization of the prefrontal cortex in the −12° head-down tilt position: A functional near-infrared spectroscopy study

**DOI:** 10.3389/fpsyg.2022.1051256

**Published:** 2022-12-21

**Authors:** Tingting Lun, Dexin Wang, Li Li, Junliang Zhou, Yunxuan Zhao, Yuecai Chen, Xuntao Yin, Shanxing Ou, Jin Yu, Rong Song

**Affiliations:** ^1^Clinical Medical College of Acupuncture, Moxibustion and Rehabilitation, Guangzhou University of Chinese Medicine, Guangzhou, China; ^2^College of TCM health care, Guangdong Food and Drug Vocational College, Guangzhou, China; ^3^Department of Traditional Chinese Medicine, Nanhai District Maternal and Child Health Hospital, Foshan, China; ^4^Department of Radiology, Guangzhou women and children's Medical Center, Guangzhou Medical University, Guangzhou, China; ^5^Department of Radiology, Southern Theater Command Hospital of PLA, Guangzhou, China; ^6^Guangdong Provincial Key Laboratory of Sensor Technology and Biomedical Instrument, School of Biomedical Engineering, Sun Yat-sen University, Guangzhou, China

**Keywords:** head down tilt, functional near infrared spectroscopy, prefrontal cortex, low dissipation optimization state, graph theory, resting-state functional connectivity

## Abstract

**Introduction:**

Our present study set out to investigate the instant state of the prefrontal cortex (PFC) in healthy subjects before and after placement in the -12°head-down tilt (HDT) position in order to explore the mechanism behind the low-dissipation optimization state of the PFC.

**Methods:**

40 young, right-handed healthy subjects (male: female = 20: 20) were enrolled in this study. Three resting state positions, 0°initial position, -12°HDT position, and 0°rest position were sequentially tested, each for 10 minutes. A continuous-wave functional near-infrared spectroscopy (fNIRS) instrument was used to assess the resting state hemodynamic data of the PFC. After preprocessing the hemodynamics data, we evaluated changes in resting-state functional connectivity (rsFC) level and beta values of PFC. The subjective visual analogue scale (VAS) was applied before and after the experiment. The presence of sleep changes or adverse reactions were also recorded.

**Results:**

Pairwise comparisons of the concentrations of oxyhemoglobin (HbO), deoxyhemoglobin (HbR), and hemoglobin (HbT) revealed significant differences in the aforementioned positions. Specifically, the average rsFC of PFC showed a gradual increase throughout the whole process. In addition, based on graph theory, the topological properties of brain network, such as small-world network and nodal degree centrality were analyzed. The results show that global efficiency and small-world sigma (σ) value were differences between 0°initial and 0°rest.

**Discussion:**

In this study, placement in the -12°HDT had a significant effect on PFC function, mainly manifested as self-inhibition, decreased concentration of HbO in the PFC, and improved rsFC, which may provide ideas to the understanding and explanation of neurological diseases.

## Introduction

HDT is a well-established simulated microgravity position, with tilt ranging anywhere between −5° and −90°. As early as 1694 HDT position has been used as a medical rehabilitation therapy for clinical diagnosis and treatment research (Kompanje et al., 2012). Under the HDT position, the autonomic nervous system is activated and subsequently modulates the cardiovascular system (Rozen et al., 2008), musculoskeletal system (Yao et al., 2013), vestibular function (Otuk et al., 2020), endocrine system (Liang et al., 2012) to protect against and adjust certain disorders associated with the autonomic nervous system. Prior studies have shown that HDT tilt larger than −4° has physiological benefits over horizontal bed rest, however, the discomfort of subjects increases at higher angles (Smith et al., 2011). Rao et al. found a decrease in vmPFC deactivation, which suggest that cerebral cortex plasticity might change after rest in −6° HDT position (Rao et al., 2014); Mathias et al. found that placement in the −12° HDT position for 21 consecutive hours might slightly reduce accuracy to some extent, but could significantly improve cognitive testing response speed, thus maintains a dynamic equilibrium in cognitive efficiency (Mathias et al., 2017). The law of conservation of energy exists in every system in nature. When one part of the system consumes less energy than the baseline, another part of the system may be improved or optimized (Schneider, 2021). For instant, hanging upside down not only allows the bats to relax completely (Lučan et al., 2009; Regnery et al., 2013), but also reduces their energy consumption and daily metabolic levels (Regnery et al., 2013). Human living systems are no exception, from this perspective, the plasticity in cerebral cortex and the dynamic equilibrium in cognitive efficiency before and after HDT position imply that the brain system may be recombined after an external stimulus.

The above discussion corresponds to the dissipative structure in the thermodynamic theory. The dissipative structure constitutes the transition channel in a system from a frustrated metastable state to another metastable state (Jeffery et al., 2019; Ueltzhffer et al., 2021), providing a new possibility to achieve energy self-optimization (Gonzalez-Ayala et al., 2020a,b). This theory is also applicable in complex biological systems. Therefore, we assume that dissipation structure like human body itself may have the capability of entering into the low-dissipation optimization state. By means of external intervention, energy consumption may be reduced, the original state may be broken by moderate self-inhibition and thus achieve a more stable and orderly state.

In order to better explore low-dissipation optimization state, we chose PFC, which is representative of the brain. The performance of PFC executive functions such as language production, attention retention, memory, and task planning is reflected in the recruitment of more neural circuits, as shown by increased neural glucose metabolism (Haier et al., 1992). Changes in neuronal activity affect the process of transporting glucose and oxygen to meet these increased metabolic needs (Bonetti et al., 2019), which in turn affects the concentration of HbO, HbR, and total HbT in the brain (Morone et al., 2017). fNIRS, an optical method for functional imaging of the brain, measures changes in HbO and HbR concentration through neurovascular coupling in the superficial layer of the brain and has been used to study neuronal responses to tasks or environmental stimuli, which is correspond to the measurement of cerebral hemodynamic changes (Pinti et al., 2020). In recent years, fNIRS has been used to measure cortical activity and functional connectivity in the resting-state brain (Han Zhang et al., 2011; Niu and He, 2014; Hu et al., 2020). Importantly, the temporal resolution of fNIRS is much higher than that of fMRI, and the technique has been widely adopted for its advantages in subject comfort, safety, portability, and minimal noise, meeting the experimental paradigm of this study.

The purpose of this study is to explore whether the PFC after short time HDT shows a low-dissipation optimization state, and to explore the corresponding relationship of low-dissipation optimization in nervous system diseases. According to previous studies, we predict that acute placement in HDT position may induce human body to make a top-down regulation from nerve to behavior, leading to the deactivation of PFC, which may inhibit thinking activity to a certain extent, adjust nerve efficiency, and recombination optimization. As one of the topological measures of complex brain networks, small-world network has been widely used in the analysis of neuroimaging and other neuroscience data for more than 20 years (Bassett and Bullmore, 2017). Previous studies have shown that topological properties of fNIRS can also be analyzed using graph theory (Niu and He, 2014; Racz et al., 2017). In order to observe more sensitively changes in neural complex network, we also adopt this method to study small-world properties (global efficiency, local efficiency, global clustering coefficient and small-world *σ* value) and nodal degree centrality (Bullmore and Sporns, 2009; Lo et al., 2011). We chose −12° HDT instead of −6° HDT, because −12° HDT has been proved to be more effective in measuring the possible correlation of the intracranial fluid system (Marshall-Goebel et al., 2016; Kramer et al., 2017). At the same time, the changes in brain hemodynamics are closely related to the situation of the intracranial fluid system (Ishida et al., 2018; Blair et al., 2020; Xiang et al., 2022). Selecting −12° HDT may be more sensitive to observe relevant changes.

## Materials and methods

### Subjects and study design

Forty healthy subjects, 20 males and 20 females (Age 20.09 ± 1.15 years, Height 165.91 ± 8.99 cm, Weight 58.18 ± 9.88 kg, Education years 13.63 ± 0.79 years) were enrolled in this study. Inclusion criteria were as follows: (1) Age 18–28 years old, (2) Right-handed subjects, (3) In good health, with clear consciousness, no hearing or cognitive impairments, and able to cooperate with the experimenter, (4) Have no history of drug or alcohol abuse, sleep disorders, or mental illness, and no consumption of caffeine or alcohol at least 12 h before the test, (5) No history of cerebrovascular disease or past cerebrovascular operations, (6) No use of sedative, anti-anxiety, or anti-depression drugs in the past month, and (7) Female subjects are non-menstrual, not pregnant, and not taking oral contraceptives. Among them, eight subjects were excluded for unstable fNIRS signals caused by significant limb movement and multiple eye openings during the experiment. Therefore, data from a total of 32 subjects (male: female = 17:15) were included for analysis. The study protocol was approved by the Ethics Committee of Nanhai Women’s and Children’s Health Hospital, Foshan, China, and each subject signed a written informed consent form prior to the trial.

### Data collection and test paradigm

#### Data collection

**Figure 1:**
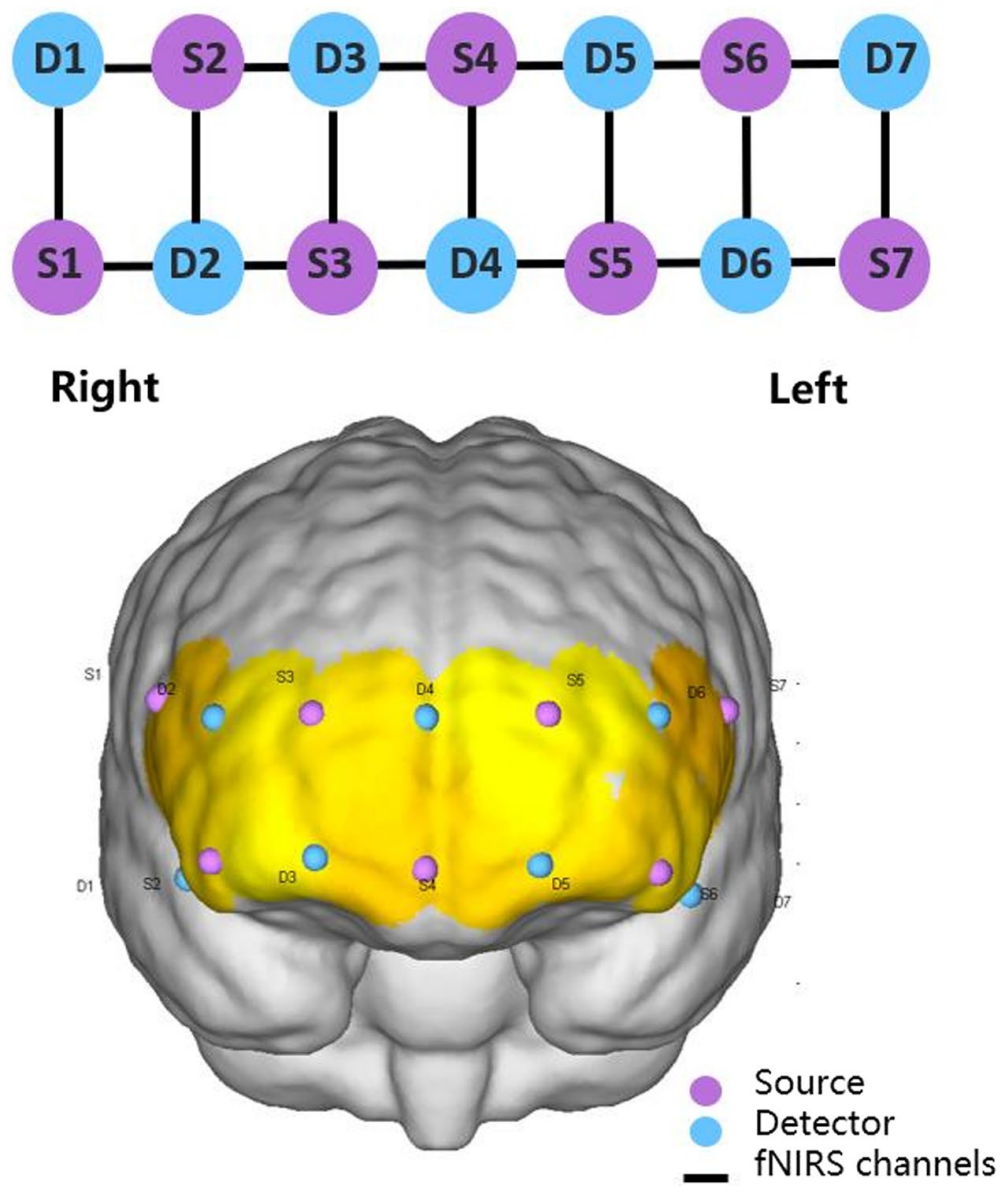
2 × 7 2D and 3D arrangement schematic diagram.

fNIRS equipment (NirSmart, Danyang Huichuang Medical Equipment Co. Ltd., China) was used for real-time measurement of continuous-wave fNIRS signals at a sampling frequency of 10 Hz. Wavelengths were set to 730 and 850 nm. Nineteen channels (defined as the midpoint of the corresponding light source-detector pair) were established, with 7 light sources and 7 detectors for measurement. The interval between adjacent probes was 30 mm, and the acquisition area of the PFC was placed in a conventional arrangement (Figure 1). The main brain regions corresponding to each channel were matched with reference to the international EEG10-20 system (Table 1). Subjects’ comfort during the −12° HDT was rated on a visual analogue scale (VAS) with numbers 0 to 10, with 0 being very uncomfortable,1 to 3 being mildly comfortable,4 to 6 being moderately comfortable, and 7 to 10 being very comfortable, no different from lying flat. Subjects were requested 5 min before and after the experiment to answer the question: “Can you estimate your comfort level during the −12° HDT on a scale with number from 0 to 10?”

**Table 1:** Percentage of main cerebral regions corresponding to each channel.

Channel	Brodmann Area	Percentage
CH1 (S1-D1)	45-pars triangularis Broca’s area	0.6760
CH2 (S1-D2)	46-Dorsolateral prefrontal cortex	0.9534
CH3 (S2-D1)	47-Inferior prefrontal gyrus	0.9594
CH4 (S2-D2)	10-Frontopolar area	0.5985
CH5 (S2-D3)	10-Frontopolar area	0.438
	11-Orbitofrontal area	0.562
CH6 (S3-D2)	10-Frontopolar area	0.9622
CH7 (S3-D3)	10-Frontopolar area	1
CH8 (S3-D4)	10-Frontopolar area	1
CH9 (S4-D3)	11-Orbitofrontal area	0.7659
CH10 (S4-D4)	10-Frontopolar area	0.9655
CH11 (S4-D5)	11-Orbitofrontal area	0.8608
CH12 (S5-D4)	10-Frontopolar area	1
CH13 (S5-D5)	10-Frontopolar area	1
CH14 (S5-D6)	10-Frontopolar area	0.988
CH15 (S6-D5)	10-Frontopolar area	0.3541
	11-Orbitofrontal area	0.6459
CH16 (S6-D6)	10-Frontopolar area	0.6163
CH17 (S6-D7)	47-Inferior prefrontal gyrus	0.9283
CH18 (S7-D6)	46-Dorsolateral prefrontal cortex	0.9319
CH19 (S7-D7)	45-pars triangularis Broca’s area	0.5298
	47-Inferior prefrontal gyrus	0.4596

#### Test environment and process

fNIRS testing was performed in an environmentally controlled laboratory with an ambient temperature of 23 ± 1°C. Keep the experimental environment dark to prevent light from interfering with fNIRS data collection (von Lühmann et al., 2019). All subjects were required to wear disposable earplugs and limit their movements – including saccadic eye movements, movement of the head, trunk, and other limbs, and speech – to avoid unnecessary motion artifacts on fNIRS (Gao et al., 2022).

The subjects will lie on a custom-made and moderate hardness flat bed with an adjustable angle (−30°~0°). The subjects will not use pillows and try to keep normal contact with the bed surface. Secure rope is used to protect the body of the subjects around the bed. The tightness should not cause discomfort to the subjects. Using the remote device to control the angle change of the bed and completing the fNIRS data marking record at the same time. TL was responsible for explaining the whole process to the subjects and informing them of the matters needing attention in waiting room. The protocol consists of three sessions, 0° initial position (supine position), −12° HDT position, and 0° rest position (supine position). Subjects first bed rest in 0° initial position, then they were transferred to bed rest at −12° HDT by remote device, and finally they were transferred to 0° rest position, both continue 10 min. DW and YX were responsible for data recording and controlling the Angle of head low with the remote control; LL and YC were in charge of asking the subjects about their feelings and performing VAS scores 10 min after the end of the experiment. In order to avoid the difference of people’s physical state due to the different time in the afternoon, we uniformly adopt the time of 9–11 in the morning for research.

**Figure 2:**
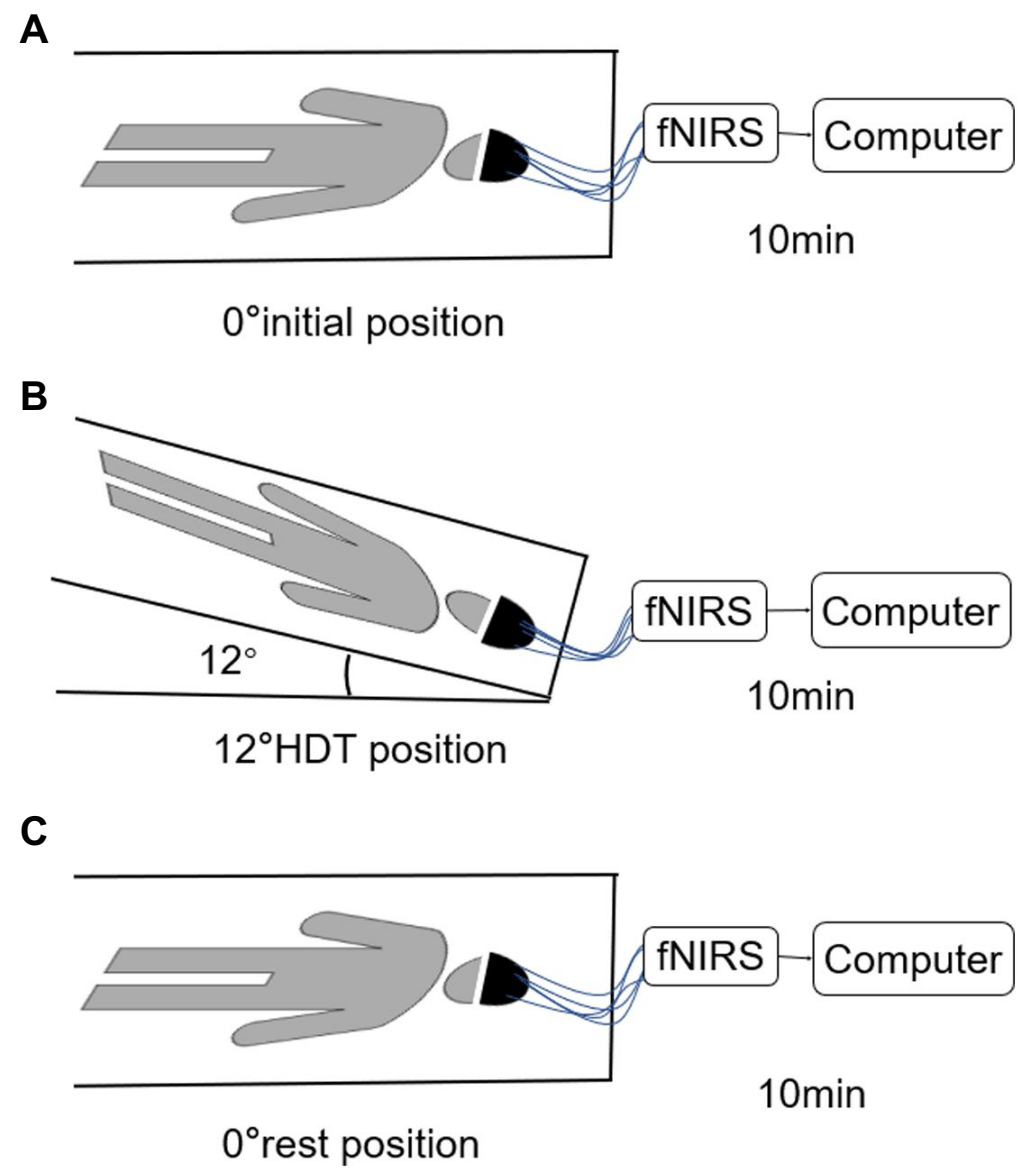
**(A–C)** are the position diagram of each stage of test paradigm.

Prior to initiating the trial, the operator explained the entire trial process to each subject until full comprehension was acquired. Subjects were placed in a supine position with their limbs positioned and their eyes closed. The researchers connected the device and adjusted the tightness and position of the electrode cap to maintain a good signal. Subjects were allowed to rest for 10 min before the test, and formal acquisition began after the signal was relatively stable (Figure 2). fNIRS data of the subjects in the 0° initial position, the −12° HDT position, and the 0° rest position was recorded continuously, and data acquisition was conducted for 10 min in each position without voice prompting during the full data collection period.

### Data processing

fNIRS data was analyzed using the NirSpark software package (HuiChuang, China), running on MATLAB2021 (Mathworks, United States). First, light intensity was converted to an optical density (OD), and any motion artifact was corrected using movement standard deviation and cubic spline interpolation (Gao et al., 2022). A band-pass filter at a frequency of 0.01–0.2 Hz was then applied to filter out the physiological noise caused by heartbeat, respiration, Meyer wave (Hu et al., 2019). Finally, the original fNIRS signals were converted into hemodynamic signals according to the modified Beer–Lambert law (Delpy et al., 1988; Kocsis et al., 2006), and concentration changes of HbO, HbR, and HbT were obtained for each channel. Data was extracted for each subject, and the HbO content in each cerebral region in the time sequence was measured (Scholkmann et al., 2014). The Pearson correlation coefficient of HbO in the time sequence of each channel and the cerebral region was calculated and defined as the rsFC strength of the corresponding channel and brain interval (Tachtsidis and Scholkmann, 2016).

GRETNA is a graph theory network analysis tool that calculates the various topological properties of a network from global and nodal characteristics (Wang et al., 2015). The Pearson correlation coefficient matrix (19 × 19) generated in the calculation based on HbT concentration will be used for further analysis in GRETNA. Since there is no clear and unified standard definition of sparsity (Wang et al., 2015), and in order to avoid error caused by a single threshold and facilitate comparison of parameters between groups, we selected 10 sparsity values of 5–50% (with an interval of 5%) to observe according to the basic settings of GRETNA. Then we binarize the absolute value of the matrix data according to the sparsity and calculate according to the related formula. In this study, the global clustering coefficient (Cp), global efficiency (Eg), local efficiency (Eloc), small-world (*σ*) value in the global small-world and nodal degree centrality (Dc) are selected for analysis. We calculated the area under the curve (AUC) for each network indicator as well as the global and nodal network indicators for each sparsity.

### Statistical analysis

All preprocessing of data was conducted in SPSS statistical software version 28.0. The data conforming to the normal distribution were described by x ± s, while those not conforming to the normal distribution were described by the median method [Md(P25, P75)]. Two-way analysis of variance was used to analyze the changes of blood oxygen, Cp, Eg, Eloc, *σ* value in the three stages; and the average of rsFC, Cp, Eg, and Eloc values were analyzed by analysis of variance (ANOVA); the non-parametric test was used to analyze σ and aDc values in three stages and paired t-tests were used to analyze the hemodynamics of men, women, and the left and right PFC. Statistical significance was defined as *p* < 0.05.

## Results

### Changes of PFC hemodynamics in different positions

**Figure 3:**
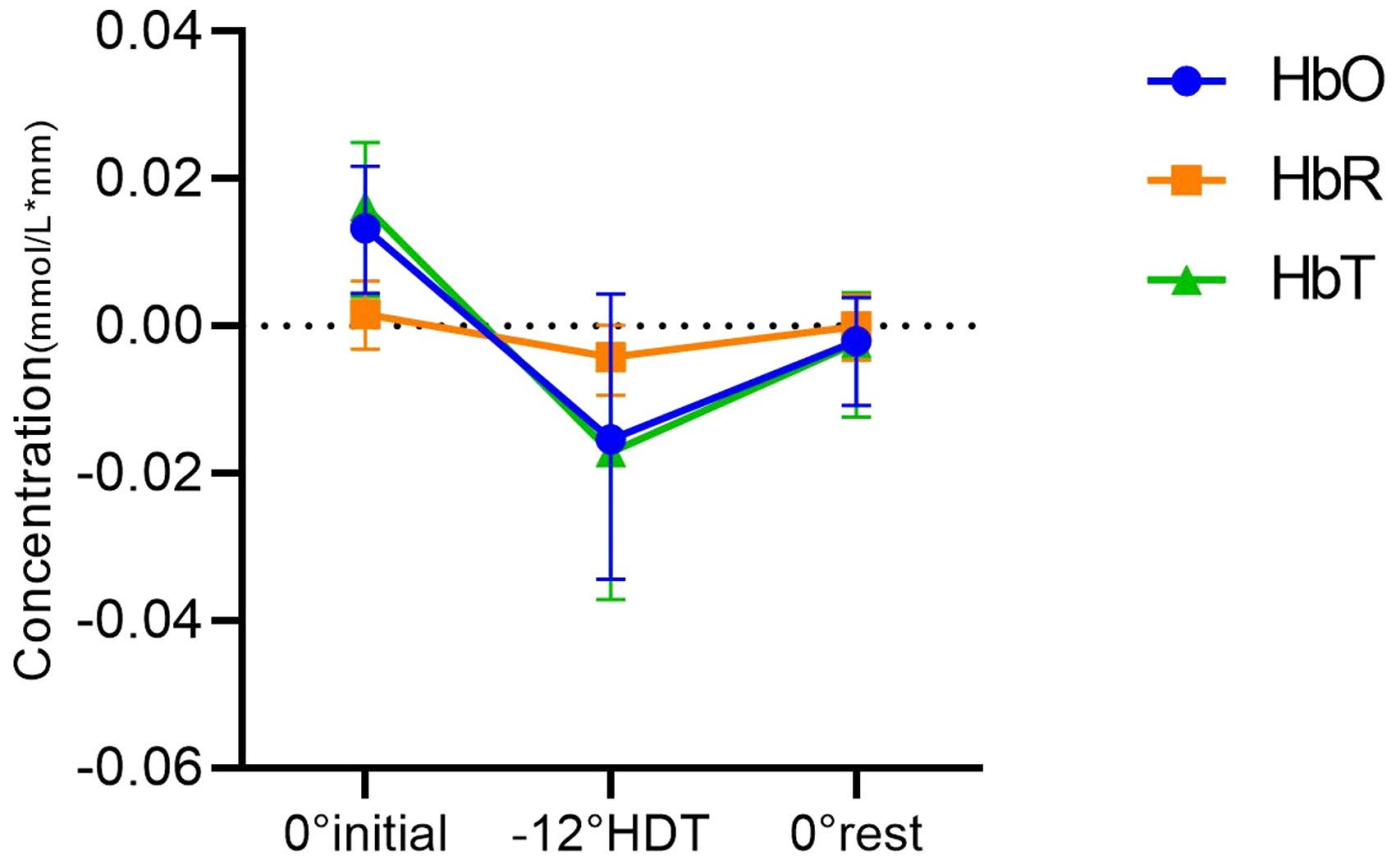
Comparison between groups of mean concentrations of HbO, HbR and HbT in different positions.

**Figure 4:**
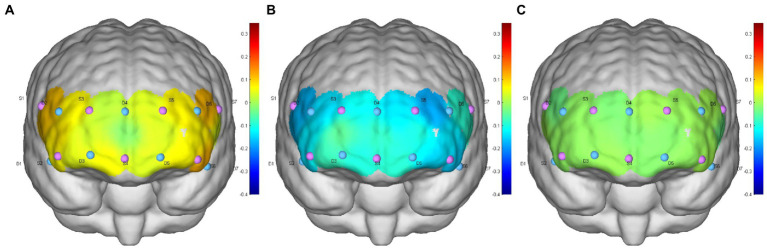
**(A–C)** are the HbO concentration distributions of subject 6 at the 0° initial, −12° HDT, and 0° rest positions, respectively.

The results of two-way ANOVA showed that 0° initial position and −12° HDT (mean difference (MD),0.026[95%CI,0.005–0.046], *p* = 0.011), 0° initial and the 0° rest position (MD,0.018[95%CI, 0.005 to 0.031], *p* = 0.006) were significantly different in HbO concentration. Nevertheless, there was no significant difference between the −12° HDT and the 0° rest (MD, −0.008 [95%CI, −0.028 to 0.013], *p* = 0.613). The change in HbT concentration between 0° initial and −12° HDT (MD, 0.031 [95%CI, 0.013 to 0.049], *p* = 0.001), 0° initial and the 0° rest (MD, 0.019 [95%CI, 0.006 to 0.032], *p* = 0.003) show significant differences. However, there was no significant difference between the −12° HDT and 0° rest (MD, −0.012 [95%CI, −0.032 to 0.008], *p* = 0.298). Changes of HbR concentration in different body positions showed no significant differences after pairwise comparisons (Table 2, Figure 3), and Figure 4 shows the changes of HbO concentration in the three stages (Figure 4).

**Table 2:** Results after pairwise comparison of hemodynamic indexes and positions.

Types	0° initial vs. −12° HDT		0° initial vs. 0° rest		−12° HDT vs. 0° rest	
	Mean Difference (mmol/L*mm)(95% CI)	*p* value	Mean Difference (mmol/L*mm)(95% CI)	*p* value	Mean Difference (mmol/L*mm)(95% CI)	*p* value
HbO	0.026 (0.005 to 0.046)	0.011	0.018 (0.005 to 0.031)	0.006	−0.008 (−0.028 to 0.013)	0.613
HbR	0.005 (−0.001 to 0.012)	0.123	0.001 (−0.004 to 0.006)	0.816	−0.004 (‘-0.009 to 0.001)	0.160
HbT	0.031 (0.013 to 0.049)	0.001	0.019 (0.006 to 0.032)	0.003	−0.012 (−0.032 to 0.008)	0.298

### Results for brain network based on graph theory

#### Global small world

**Figure 5:**
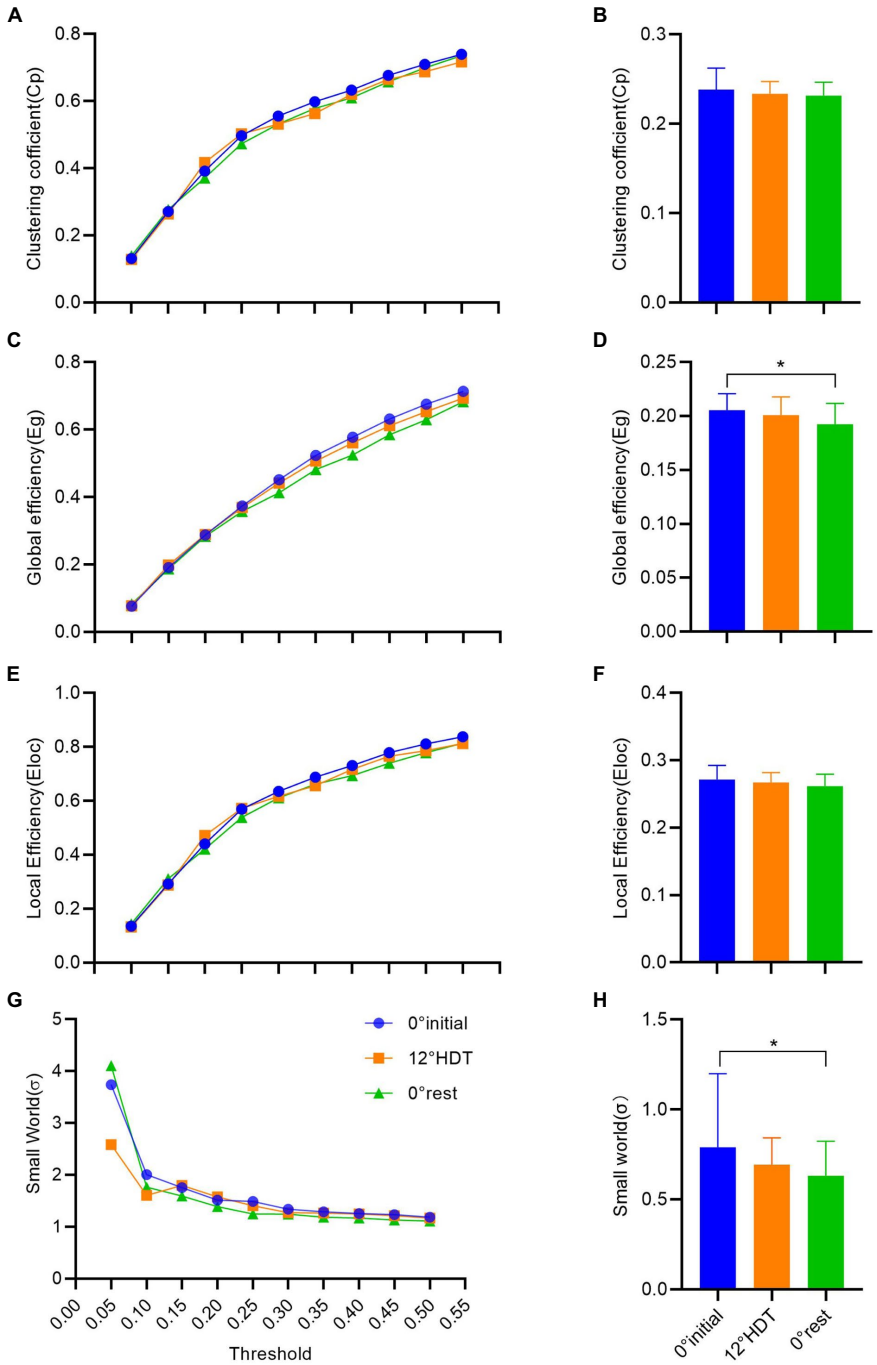
**(A–H)** successively shows the analysis results of clustering coefficient, global efficiency, local efficiency and σ value in the small world of complex brain networks based on graph theory. (*p < 0.05 and **p < 0.01).

In the clustering coefficient of global small-world network, the C*p* values increased as the thresholds increased. There was no significant difference in C*p* values under different thresholds except for the comparison between the thresholds of 0.40 and 0.45 (MD, −0.032 [95%CI, −0.068 to 0.004], *p* = 0.103) and 0.45 and 0.50 (MD, −0.032 [95%CI, −0.067 to 0.004], *p* = 0.105), and the rest are all different (Figure 5A). However, there was no significant difference [*F* (2, 93) = 1.126，*p* = 0.329] and the overall clustering coefficient showed a tiny decline in three positions (Figure 5B).

The Eg values also increased with the increase of thresholds, and the comparison between Eg values under different thresholds was significantly different [*F* (9, 18) = 1,029, *p* < 0.001]. The comparison of Eg values at different positions under each threshold showed that there was no significant difference between −12° HDT (MD, 0.010 [95%CI, −0.002 to 0.023], *p* = 0.129) and 0° initial and 0° rest (MD, 0.28 [95%CI, 0.015 to 0.040], *p* < 0.001) and −12° HDT and 0° rest (MD, 0.017 [95%CI, 0.005 to 0.030], *p* = 0.007; Figure 5C). The overall Eg values of three stages was significantly different at 0° initial and 0° rest (MD, 0.013 [95% CI, 0.002 to 0.024], *p* = 0.013; Figure 5D). The Eloc values also increased with the increase of thresholds. The Eloc values at different thresholds at the three positions did not show significant differences when the thresholds were 0.40 and 0.45 (MD, −0.031 [95%CI, −0.075 to 0.014], *p* = 0.715) and 0.45 and 0.50 (MD, 0.030 [95%CI, −0.074 to 0.015], *p* = 0.859). The Eloc values at the other thresholds all showed significant differences (*p* > 0.05; Figure 5E). The overall Eloc values did not differ significantly between three stages [*F* (2, 93) = 2.403, *p* = 0.096] and showed a tiny decrease (Figure 5F).

In the small-world *σ* value, *σ* values decreased as the thresholds increased. Except for the significant difference between the *σ* values under the threshold of 0.05 and *σ* values of other thresholds under three positions, the pairwise comparison of the other *σ* values under different thresholds showed no significant difference (*p* < 0.001;Figure 5G). The overall σ values of 0° initial was significantly different from 0° rest (adjusted *p* = 0.024), and there was a tiny downward trend in the three stages (Figure 5H).

#### Nodal degree centrality

**Figure 6:**
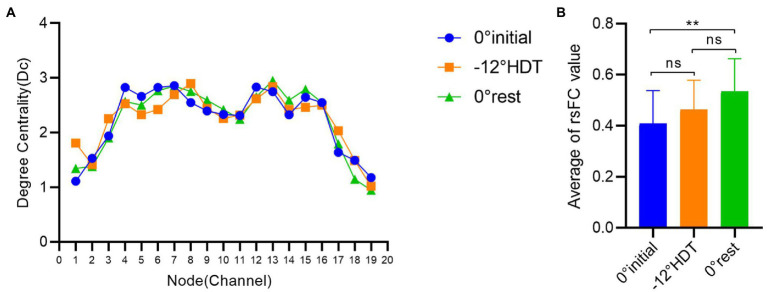
**(A)** Comparison of degree centrality of different nodes in three stages, **(B)** Comparison of brain functional connectivity values between groups in different positions. (*p < 0.05 and **p < 0.01).

In terms of the degree centrality of nodes, there exists no significant difference between the Dc values of three stages under different nodes (*p* > 0.05), among which the Dc values of nodes 4 to 16 are larger than those of other nodes, and the overall trend shows a roughly symmetric trend with nodes 10 and 11 as the central axis (Figure 6).

### Results for rsFC values

**Figure 7:**
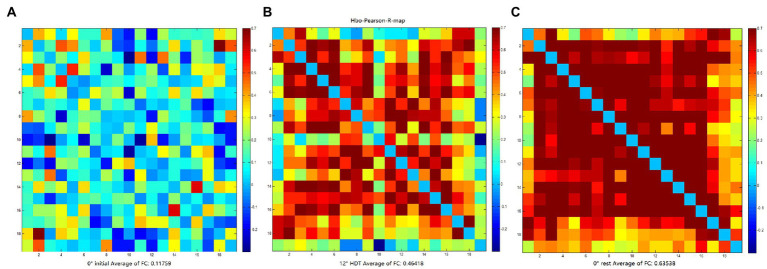
**(A–C)** are FC matrix plots for subject 6 in the 0° initial, −12° HDT, and 0° rest positions, respectively, showing a significant increase in functional connectivity from channel 3 to channel 16.

The rsFC is considered to a general representation of functional brain structures across cognitive functions and individual differences (Niu and He, 2014; Tachtsidis and Scholkmann, 2016). Statistically significant differences were identified in the overall mean rsFC values in different positions (*F* = 8.169, *p* = 0.001), in particular the 0° initial and 0° rest position (*p* = 0.000). The rsFC values of the 0° initial and − 12° HDT (*p* = 0.238) and −12° HDT and 0° rest position (*p* = 0.079) showed no significant difference. However, with the changes in three positions, the rsFC of the brain was gradually enhanced, particularly channel 3 to channel 16 (Figure 7).

### Comparison of other variable factors

HbO concentrations in the left and right PFC showed no significant differences when compared in the 0° initial (*t* = 0.655, *p* = 0.517), −12° HDT (*t* = −0.535, *p* = 0.596), and 0° rest position (*t* = −0.268, *p* = 0.791; Table 3). The rsFC values between male and female subjects in 0° initial (*t* = 1.484, *p* = 0.148), −12° HDT (*t* = 0.457, *p* = 0.652), and 0° rest position (*t* = 1.261, *p* = 0.217) showed no significant differences (Table 4).

**Table 3:** Comparison of HbO concentrations in the left and right PFC in different positions.

Position	Mean ± SD (×10^−4^ mmol/L*mm)		*t*	*p*
	Left	Right		
0° initial	7.67 ± 2.16	6.81 ± 2.05	0.655	0.517
−12° HDT	6.86 ± 3.64	6.05 ± 3.60	−0.535	0.596
0° rest	2.24 ± 2.10	2.07 ± 1.76	−0.268	0.791

**Table 4:** Comparison of rsFC values between men and women in different positions.

Position	rsFC value (Mean ± SD)		*t*	*p*
	Male (*n* = 17)	Female (*n* = 15)		
0° initial	0.44 ± 0.13	0.37 ± 0.12	1.484	0.148
−12° HDT	0.47 ± 0.15	0.45 ± 0.07	0.457	0.652
0° rest	0.56 ± 0.13	0.50 ± 0.12	1.261	0.217

### VAS score and adverse reactions

There was no significant difference in VAS scores before and after the experiment (t = −0.643, *p* = 0.526; Pre-test Mean = 6.67, Post-test Mean = 6.7). There were no serious adverse reactions during the experiment. Five subjects fell asleep slightly, and the remaining subjects did not fall asleep. Most subjects experienced head bulges when placed in the −12° HDT position, but they recovered quickly after restoration to the 0° rest position. One subject had a mild headache prior to the trial, but the headache resolved after placement in the −12° HDT position.

## Discussion

### The PFC showed self-inhibition effect and delay effect

In this study, −12° HDT may induced self-adaptive changes in PFC and a reduction in the concentration of HbO. There was no significant difference in blood oxygen status between the 0° rest position and the −12° HDT after recovery, showing a position delayed effect. Cooke et al. previously found that in the acute HDT position dynamic and automatic cerebral regulation is normal when the body position is changed (Cooke et al., 2003). Meanwhile, rapid changes of body position can affect blood perfusion in the head and the stimulation of arterial baroreceptors, causing bottom-up cortical inhibition (Lipnicki, 2009), activating the synergistic effects of circulatory-cortical homeostasis (Pollatos et al., 2010). The process of the functional hyperaemia is a basic feature of microcirculation, which depends on neurophysiological metabolism and vascular involvement. Research has shown that many psychiatric degenerative diseases may be caused by the lack of synergistic ability between neurovascular coupling (Iadecola, 2004). Temporarily adjust the circulation of PFC by short-term HDT head position may provide an opportunity to promote the release of active factors associated with neurovascular coupling.

Meanwhile, based on the graph theory analysis, the greater global network efficiency is, the higher information transmission efficiency will be between networks (van den Heuvel and Hulshoff Pol, 2010; Bullmore and Sporns, 2012). However, in this study, 0° rest slightly decreased compared with 0° initial, which may indicated that the network information transmission efficiency was self-inhibited effect. This moderately self-repression results in sympathetic nerve activity decreased (Birch et al., 1995; Cooke et al., 2003) may correlated with the Cp, Eg, Eloc, and *σ* values decreased slightly after −12° HDT. Previous studies have also shown that apathy and inhibition states as measured by the frontal system behavior scale are positively correlated with Eg and Cp value (Reyes et al., 2018). Furthermore, the neuro efficiency hypothesis reflects that getting higher score on intelligence tests individuals show lower (more effective) brain activation in performing cognitive tasks (Neubauer and Fink, 2009). The decrease of BOLD signal and HbO may be due to the shunting of blood from less active areas to areas that require the most cerebral blood flow (Harel et al., 2002), as well as focal inhibition of neural activity (Fransson, 1999; Raichle et al., 2001). Therefore, the −12° HDT may reduced energy expenditure in the PFC and have greater activation and improvement in other brain regions.

Nevertheless, there are still some indicators in this study that need to be carefully interpreted. The Cp value is generally regarded as an indicator of the information processing efficiency of the local brain regions of the brain network (Masuda et al., 2018); and the Eloc value is the main index to evaluate the fault-tolerant rate of network information transmission (Achard and Bullmore, 2007). However, there was no significant difference in these indexes in three stages. But studies have shown that the brain uses only a fraction of the energy of neurons in the resting state to adjust their functions (Raichle and Mintun, 2006)，we speculated that −12° HDT period was relatively short, which did not cause relevant changes and triggered fully. The small-world σ value is the ratio of the clustering coefficient to the average path length. In this study, all the σ values under different thresholds are all greater than one (Hlinka et al., 2012), indicating that the network belongs to the small-world network, that is, the network has a short wiring cost and a high transmission efficiency. However, because the σ value is greatly affected by network scale (Hlinka et al., 2012), which needed to be interpreted with caution.

### The −12° HDT enhanced rsFC values

The rsFC of PFC increased after placement in the −12° HDT, indicating that enhanced connectivity of various parts of PFC helped to process information more flexibly when the brain might be in a state of adapting in conjunction with the body’s related systems. The most obvious changes were observed in the inferior frontal gyrus, orbitofrontal cortex and frontopolar region, which are related in cognitive-affective processing (Rudebeck and Rich, 2018; Heather Hsu et al., 2020). Studies have shown that certain patient populations, such as elderly people suffering from major depression (Solomonov et al., 2020), people with related cognitive impairment (Srivastava et al., 2021), and migraine patients (Argaman et al., 2020) have low rsFC in corresponding parts of the brain. There is also a phenomenon of unilateral entropy increase (Akdemir Akar et al., 2015), also known as a specific nonlinear disorder (Haken, 1973). This is consistent with our previous hypothesis of low-dissipative optimization of the PFC. Dissipative structure in system sciences (Nakamura et al., 2021) is comparable to the work of the brain: on the one hand, the brain in a low dissipation state can regulate the entire energy consumption, which is essential for body resting and recovering; on the other hand, any change interfering brain order would probably lead to overall recombination and optimization (Gonzalez-Ayala et al., 2020b).

### The effects of HDT on left and right PFC and gender were not obvious

We further analyzed the results according to gender and the outcome of the corresponding analysis of the left and right PFC for each position. Previous studies have shown that lateralization occurs in people with large portions of the brain under normal conditions (Güntürkün and Ocklenburg, 2017; Güntürkün et al., 2020), but it did not conform to our experiment, and the neural mechanisms involved are still unclear; In addition, gender differences generally affect the adaptation of innervated cerebral blood flow (Hines, 2020; Ristori et al., 2020), yet our experiment showed that there was no significant change in HbO changes between male and female at 3 positions, which may indicate that there may be a common balance mechanism in the human body to control each other. The VAS score proved that there has been no significant difference and the comfort level has been moderate, indicating that −12° HDT for 10 min was acceptable and has not caused discomfort.

### The association between low-dissipation optimization state and mental disorders

The greater nodal degree centrality is, the more important nodes will be in the network (Rubinov and Sporns, 2010). Nodes correspond to the brain regions represented by channels, and this study shows that frontopolar area and orbitofrontal area played major roles. Mental disorders diseases often have abnormal brain function. For example, the average functional connectivity (FC) measured from patients with bipolar affective disorder and major depressive disorder under stress is significantly reduced (Takizawa et al., 2008); Task-related changes in HbO in PFC of patients with depression were not as significant as in healthy individuals，the FC of right inferior frontal gyrus and orbitofrontal cortex is lower (Takizawa et al., 2008; Zhu et al., 2017; Rolls et al., 2020). In addition, regions such as the frontal cortex are shown to decrease in size due to abnormal FC associated with cognitive impairments in schizophrenia patients (Sheffield and Barch, 2016). Studies have also shown abnormal activation in the anterior cingulate cortex and orbitofrontal cortex and hyperactivation (Kumar et al., 2017) of the right frontal cortex in schizophrenic patients during task tests. If the abnormal activation can be suppressed at that time, it may be possible to bring the brain state back into balance. Thus, we have noticed that patients with psychiatric disorders often suffer from abnormal brain state. It would be considerable if we could adjust and optimize the corresponding brain lesion area with the idea of low-dissipation optimization state, so as to regulate the abnormal FC of brain and adjust the efficiency and optimization of the brain by moderate inhibition of the PFC.

## Conclusion

In general, from the perspective of thermodynamic dissipative structure theory and biomimetic science, the −12° HDT position can reduce the oxygen content and enhance rsFC through the reduction of the global efficiency of PFC, thus leaving putting PFC in a low-dissipation optimal state. To analyze topological properties of complex brain networks based on graph theory can deepen the research from another angle. Inhibition followed by some conscious guidance has the potential to increase brain orderliness, and neural low-dissipation optimization provides insights into the understanding and explaining neurological diseases.

## Areas for improvement

Due to condition limits, we were unable to include ample subjects, leading to small sample size. Future experiment should systematically assess a greater range of HDT angles and drill down on the low-dissipation optimal state. Additionally, there may be physiological phenomenon of increased intracranial pressure at HDT position (Taibbi et al., 2013), which may cause intracranial substances (such as cerebrospinal fluid, brain tissue, etc.) to affect the data of the measured site. If more brain areas are used for measurement in the future, this problem should be noted, or more advanced algorithms should be developed to solve it. Our next steps will be to collect hemodynamic data in different regions of the brain at the same time, expand our sample size, as well as include patients with depression or anxiety symptoms.

## Data availability statement

The data generated and analyzed during the current study is not publically available for legal/ethical reasons. This data can be made available by the corresponding author/s upon reasonable request.

## Ethics statement

The studies involving human participants were reviewed and approved by Committee of Nanhai Women’s and Children’s Health Hospital (no. 2021-03), Foshan, China. The patients/participants provided their written informed consent to participate in this study. Written informed consent was obtained from the individual(s) for the publication of any potentially identifiable images or data included in this article.

## Author contributions

Under the guidance of JY and RS, TL and DW designed the study and wrote the manuscript. LL conducted the experimental operation and informed consent writing. JZ, YZ, and YC participated in the data acquisition and critical revision of the manuscript. SO and XY provided the methods for fNIRS analysis. All authors provided important feedback and helped shape the study, analysis, and manuscript.

## Funding

The authors received the following financial support for the research, author status, and/or publication of this article: (1) Guangdong University Scientific Research Platform and Project of Education Department of Guangdong Province (no. 2019KZDZX1041); (2) Guangdong Provincial Key Laboratory of Sensor Technology and Biomedical Instrument (no. 2020B1212060077); (3) Guangdong Traditional Chinese Medicine Health Service and Industry Development Research Center Project (no. 2022ZDA01).

## Conflict of interest

The authors declare that the research was conducted in the absence of any commercial or financial relationships that could be construed as a potential conflict of interest.

## Publisher’s note

All claims expressed in this article are solely those of the authors and do not necessarily represent those of their affiliated organizations, or those of the publisher, the editors and the reviewers. Any product that may be evaluated in this article, or claim that may be made by its manufacturer, is not guaranteed or endorsed by the publisher.
